# Correlation between lumbar dysfunction and fat infiltration in lumbar multifidus muscles in patients with low back pain

**DOI:** 10.1186/s12891-016-1376-1

**Published:** 2017-01-10

**Authors:** Markus Hildebrandt, Gabriela Fankhauser, André Meichtry, Hannu Luomajoki

**Affiliations:** 1 Physio Hildebrandt, Sickingerstrasse 4, 3014 Bern, Switzerland; 2 Hauptstrasse 26, 3254 Messen, Switzerland; 3 Institute of Physiotherapy, School of Health Professions, Zurich University of Applied Sciences, Technikumstrasse 71, 8401 Winterthur, Switzerland

**Keywords:** Low back pain, Multifidus muscle, Fat infiltration, Flexibility

## Abstract

**Background:**

Lumbar multifidus muscles (LMM) are important for spinal motion and stability. Low back pain (LBP) is often associated with fat infiltration in LMM. An increasing fat infiltration of LMM may lead to lumbar dysfunction. The purpose of this study was to investigate whether there is a correlation between the severity of lumbar dysfunction and the severity of fat infiltration of LMM.

**Methods:**

In a cross-sectional study, 42 patients with acute or chronic LBP were recruited. Their MRI findings were visually rated and graded using three criteria for fat accumulation in LMM: Grade 0 (0–10%), Grade 1 (10–50%) and Grade 2 (>50%). Lumbar sagittal range of motion, dynamic upright and seated posture control, sagittal movement control, body awareness and self-assessed functional disability were measured to determine the patients’ low back dysfunction.

**Results:**

The main result of this study was that increased severity of fat infiltration in the lumbar multifidus muscles correlated significantly with decreased range of motion of lumbar flexion (*p* = 0.032). No significant correlation was found between the severity of fat infiltration in LMM and impaired movement control, posture control, body awareness or self-assessed functional disability.

**Conclusion:**

This is the first study investigating the relationship between the severity of fat infiltration in LMM and the severity of lumbar dysfunction. The results of this study will contribute to the understanding of the mechanisms leading to fat infiltration of LMM and its relation to spinal function. Further studies should investigate whether specific treatment strategies are effective in reducing or preventing fat infiltration of LMM.

## Background

Low back pain (LBP) has a very high incidence rate with a lifetime prevelance of up to 84% [1]. Persisting pain for more than 12 weeks is defined as chronic low back pain (CLBP) [1]. Most LBP disorders are multifactorial in nature and there are diverse interpretations for the underlying pain mechanisms, even when specified radiological diagnosis are found. Eighty-five percent of CLBP disorders have no specific diagnosis or pathology and are therefore “nonspecific” [1]. A large group of these disorders are predominantly mechanically induced and lead to maladaptive processes that maintain the ongoing pain and can result in functional deficits [2]. There is evidence that persisting LBP influences lumbar motor control [3], alters brain function and structure [4], changes lumbar tactile acuity [5], decreases spinal mobility [6] and compromises postural control [7]. However, LBP does not only lead to dysfunction, it can also result in structural changes of the lumbar multifidi muscle (LMM) such as fat infiltration as a consequence of atrophy [8–10].

Lumbar multifidus muscles are important for providing segmental stability and they function as dynamic stabilizers of the lumbar spine. They reinforce lumbar lordosis during rotation [11] and antagonize lumbar flexion [41]. It is generally assumed that dysfunction of the back muscles results in pain inhibition, which can finally lead to fatty infiltration of the LMM [9, 12]. Additionally, metabolic [13] or neuropathic mechanisms [14] are possible causes for the appearance of muscle degeneration. The average fat content of LMM in healthy subjects is down to levels as low as 14.5%, whereas in subjects with CLBP, the fat content of LMM can average levels as high as 23.6% [15, 16]. Interestingly, there is no correlation between obesity and the presence of fat in LMM [16]. About 80% of people suffering from CLBP present LMM with increased fat infiltration between levels L2 to L5 [10, 16, 17]. Increased fat infiltration in association with pain, age or dysfunction has also been reported in other muscles [18–20]. Association between chronic neck pain, fatty infiltration of sub-occipital muscles [21] and dysfunction of standing balance [22] could be indicated. For the lower back, the relationship between sway-back posture and a greater fat deposition in LMM could be demonstrated [23]. However, further studies that investigate the association between fat infiltration and dysfunction of the lower back are missing.

The purpose of this study was to seek possible correlation between fat infiltration of LMM and specific lumbar dysfunction in patients suffering from LBP. Based on the fact that LMM are important for motion and stability of the spine, we hypothesized that fat infiltration of LMM could be associated with impaired movement and posture control.

## Methods

This cross-sectional study was conducted in a private physiotherapy outpatient clinic in Bern, Switzerland, according to the Helsinki declaration of ethics in medical research. The duration of the study was 8 months (May-December 2013).

### Participants

Forty-two patients with non-specific LBP and a referral for physiotherapy, who had a recent magnetic resonance imaging (MRI) of their lower spine, were consecutively recruited for this study from different healthcare centers. Patients were not allowed to be familiar with the measures used in this study and they should not have received manual therapy or lumbar stabilization programs prior to this study. Patient data for age, gender, body weight and duration of LBP (acute pain < 12 weeks, chronic pain > 12 weeks) were collected and the body mass index (BMI) for each patient was documented. Patients who were obese (BMI > 35) or not able to perform active lumbar flexion and extension due to pain inhibition were excluded from this study. Those who had prior back surgery, sacroiliac arthritis, acute lumbar trauma, neurological deficits, active malignancy, infectional diseases, and were under the age of 20 or over the age of 75 were excluded as well.

### MRI evaluation

The patients’ MR images were generated prior to this study for medical diagnostics and not for the purpose of this study. MR images were obtained with 1.5 T systems (GE Medical Systems, USA; Siemens Healthcare, Erlangen, Germany) and patients were positioned supine in the MRI device. Each MRI sample contained standard T1- and T2-weighted axial images of the lumbar spine. All images were stored as DICOM format for processing. Due to the clinical setting of this study, we had to accommodate the fact that MR images were produced in different radiology centers and MRI parameters were not standardized. Analyze software (OsiriX, Pixmeo SARL, Switzerland, Version 5.6) was used for image analysis. To determine fat infiltration of the lumbar multifidus muscles, all axial T1-weighted MR images were included in the analysis as such sequences provide excellent anatomical detail.

While many quantitative MRI-based methods like Dixon/IDEAL [24, 25] or proton-density fat-fraction [26] are more accurate to measure fat separation, we had to take the variable MRI parameters into account. Semiquantitative assessment of fat infiltration of lumbar muscles have been reported to be valid and reproducible, and findings correlated with MR spectroscopic measurements [15]. There is also evidence that visual grading of fat infiltration in LMM, using MR images, is reliable [27, 28]. For that reason, a visual evaluation method was used for this study.

**Fig. 1:**
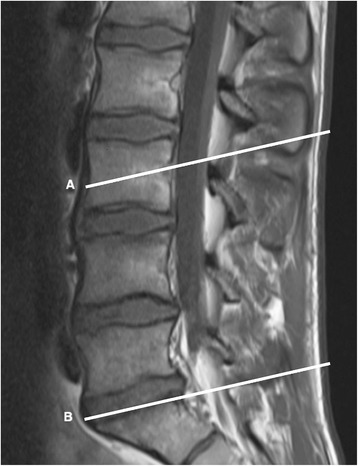
Sagittal view depicts the range (A-B) within axial MR images were analyzed

**Fig. 2:**
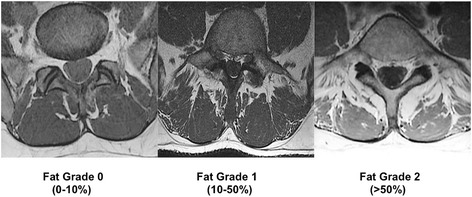
Grading of MR images with different muscle-fat compositions of lumbar multifidus muscle. The slice demonstrating the highest quantity of fat infiltration was graded accordingly

Images were analyzed slice per slice within the determined range between L3 and L5 (Fig. 1). In order to optimize image quality, grey scaling was used during analysis. The area demonstrating the highest quantity of fat infiltration in LMM (left and right side combined) was used for grading. To determine fat infiltration of LMM three criteria were used (Fig. 2): Grad 0 (0–10% fat), Grade 1 (10–50% fat) and Grade 2 (>50% fat) [16].

Grading for this study was performed by a doctor of chiropractic who is a clinician and instructor of radiological diagnostics at the national chiropractic academy with 25 years of experience. He was blinded to the patients functional assessments.

### Measures

Lumbar dysfunction was specified as reduced spinal flexibility, impairment of movement and posture control, attenuation of body awareness and self-assessed functional disability. In order to assess the patients’ extent of LBP and the functional abilities of the low back, a set of different tests were used. All tests were performed by the same investigator, a physical therapist with 20 years of experience in manual therapy. To minimize testing bias, procedures were standardized and trained prior to the study. Patients wore only underwear to allow the observation of the entire body. The investigator was not aware of the patients’ MRI findings.

#### Lumbar flexibility

Measurement of spinal flexibility was performed with the Spinal Mouse®, a hand-held computer-assisted device that can be used to measure the global and segmental range of motion of the spine [29]. The Spinal Mouse® has acceptable metrological properties to assess segmental and global lumbar flexibility during trunk flexion. However, its metrological properties are not acceptable to assess segmental mobility of L5-S1 alone [30] and the segmental mobility of obese persons. Patients active range of motion of lumbar flexion (L1-S1), lumbar extension (L1-S1) and hip flexion were recorded in this study. Prior to the measurements, the landmarks of C7 and S3 were determined by palpation and were marked with a waterproof marker.

The device was then guided between the landmarks and along the midline of the spine to conduct the measurement.

#### Postural control

**Fig. 3:**
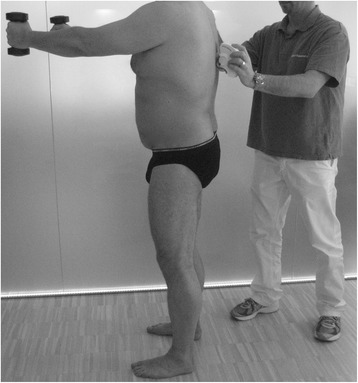
Measurement of upright Matthiass’ arm-raising test with the Spinal Mouse®. Segmental and total evasive movements for flexion or extension of the spine were calculated based on the difference between pre- and post-test posture

Postural control is based on the regulation of multisensory inputs and the reactions to stabilization. For this study, the upright Matthiass’ arm-raising test [31] was used. This is a clinical test to detect posture changes under dynamic conditions. Patients had to hold two dumbbells with extended arms at shoulder height while posture was measured twice within an interval of 30 s with the Spinal Mouse® (Fig. 3). The overall weight of the dumbbells was calculated according to gender and bodyweight (women 5%, men 6.5% of bodyweight). Total and segmental evasive movements for the lumbar spine were calculated based on the differences between the two measurements.

#### Movement control

Movement control tests (MCT), a test battery consisting of six tests, are a reliable instrument for evaluating the ability to control flexion, extension and rotation of the lower back [3]. For practical reasons, MCT were modified for this study and only four tests were conducted to assess flexion and extension control (waiters bow, pelvic tilt, seated knee extension and prone active knee flexion). Patients received standardized instructions and each test was rated after three attempts. A correct movement (test negative) was rated with zero and an incorrect movement (test positive) was rated with one point. Total scores (max. four points) for all patients were calculated.

#### Body awareness

Impaired movement control of the lower back correlates positively with a disruption of the body image measured by two-point discrimination (TPD) of the back [5]. TPD threshold was measured using a plastic calliper ruler in the area between the first lumbar vertebra and the iliac crest. The threshold was defined as the distance between the calliper points at which the participants could decidedly detect two points instead of one. To find the TPD thresholds, descending runs with 10 mm increments starting from 8 cm as well as ascending calibrations with 5 mm increments were used. TPD was measured in prone position, left and right from the midline of the spine, horizontally and vertically. Mean values and standard deviations were calculated for horizontal and vertical thresholds.

#### Functional disability

To assess the implication of LBP on daily activities, the German version of the Oswestry Disability Index (ODI) questionnaire was used. The self-administered ODI is a valid instrument for measuring the degree of disability and for outcome measurement [32]. The German version of the ODI has been validated [33]. For this study the ODI was modified by using only nine sections, with a score ranging from 0 – 5 points per section. Total scores for all patients were calculated.

### Statistical analysis

For each response, we fit a linear model to the data using fat, gender, age, duration of LBP and BMI as covariates. Our linear model for the *j*-th subject from group *i* was *Y*_*ij*_ *= μ + α*_*i*_ + covariates _*ij*_ *+ ε*_*ij*_, with *μ* as the intercept, *α*_*i*_ as the fat-effect of group *i* and the *ε*_*ij*_ as independent and normally distributed errors. We were interested in the covariate-adjusted effect of fat on the outcomes. Pairwise contrasts between the fat-groups were estimated from the estimated model. All simultaneous inference procedures controlled the family-wise error rate of *α* = 0.05. Residual analysis was performed to check model assumptions, that is, independent and normally distributed errors. Breusch-Pagan tests were performed to test for homogeneous variances and Shapiro-Wilk tests for the normality assumption. For the association between fat content of LMM and age, status, gender and BMI, the Pearson correlation coefficient was calculated. A *P* value of less than .05 was considered to demonstrate a statistically significant difference.

The statistical analysis was performed with R, version 3.1.0 [34].

## Results

A total of 42 patients, nineteen women (47.21 ± 13.15 years of age, 21–71 years) and 23 men (40.35 ± 10.21 years of age, 22–62 years) were tested. A detailed delineation of physical characteristics of the cohort is displayed in Table 1.

**Table 1:** Physical characteristics in participating patients. Values for age and body mass index (BMI) represent mean and standard deviation

Fat Grade	Grade 0	Grade 1	Grade 2
Participants (*n* = 42)	*n* = 6	*n* = 25	*n* = 11
Gender (male, female)	6, 0	15, 10	2, 9
Duration of pain (acute, chronic)	4, 2	8, 17	1, 10
Age (years)	36 (10.49)	41.92 (11.60)	51 (10.50)
Body mass index (kg/m2)	25.24 (2.51)	23.53 (3.53)	22.49 (2.61)

The results of main measurements are listed in Table 2.

**Table 2:** Descriptive data of main outcomes. Increased fat content of multifidus muscle correlates with decreased lumbar flexion (*p* = 0.032)

Fat Grade	Grade 0	Grade 1	Grade 2
Lumbar flexion (degrees)	24 (9.72)	22.56 (11.62)	11.55 (12.49)
Posture control (degrees)	–2.33 (1.37)	–1.08 (1.63)	–0.73 (0.90)
Movement control score (0–4)	2.17 (0.75)	1.68 (1.07)	1.18 (0.87)

Data represent mean and standard deviation. Negative values represent evasive movement in lumbar extension

69.1% of the patients reported chronic LBP and 30.9% reported acute LBP. Almost 85% of the patients showed fat infiltration in their LMM. Patients with chronic LBP were more likely to have fatty infiltration in LMM than patients with acute LBP (*p* = 0.043). Female patients demonstrated more fat infiltration in LMM than male patients (*p* = 0.0019), with a striking difference in fat grade 2. Whereas age correlated significantly with the presence of fat infiltration in LMM (*p* = 0.025), patients BMI did not interfere with fat infiltration in LMM.

### Relationship between impairments and fat grade

Results showed that increased severity of fat infiltration in the lumbar multifidus muscles correlated with decreased range of motion of lumbar flexion (*p* = 0.032). Pairwise contrasts between the fat-groups indicated a significant difference (*p* = 0.039) between fat Grade 1 and fat Grade 2 (12.42°, 95% CI 0.513, 24.3) (Table 3).

**Table 3:** Table shows the estimated contrasts for lumbar flexion between the fat grades with the corresponding standard error (SE), degrees of freedom (df), t-statistic (t), the *p*-value (p) and the lower and upper bound of the 95% confidence interval (uCL and lCL)

Contrasts	Estimate	SE	df	ICL	uCL	t	*p*
Grade 0 - Grade 1	1.83	5.83	35	-18.35	22.0	0.315	0.947
Grade 0 - Grade 2	14.26	7.57	35	-11.93	40.4	1.885	0.158
Grade 1 - Grade 2	12.42	4.87	35	-4.42	29.3	2.553	0.039

The results are averaged over the levels of gender and status and taken at the mean of age and BMI. The *p*-values are adjusted for multiple testing. Results are averaged over the levels of: Gender, Status. Confidence level and *P*-value adjustments: tukey method for a family of 3 means.

However, none of the effect moderators (age, gender, duration of LBP, BMI) correlated with lumbar flexion (Table 4).

**Table 4:** ANOVA table for the effect of fat grade and the covariates on lumbar flexion

Parameters	Df	SS	MS	F	*P*
Fat	2	1046	523	3.8	0.032
Age	1	72	72	0.52	0.474
Gender	1	54	54	0.39	0.535
Duration of pain	1	279	279	2.03	0.164
Body mass index	1	48	48	0.35	0.561
Residuals	35	4820	138		

*Df* Degrees of freedom; *SS* Sum of squares, *MS* Means squares, *F* F-statistics, *p* *p*-value

**Fig. 4:**
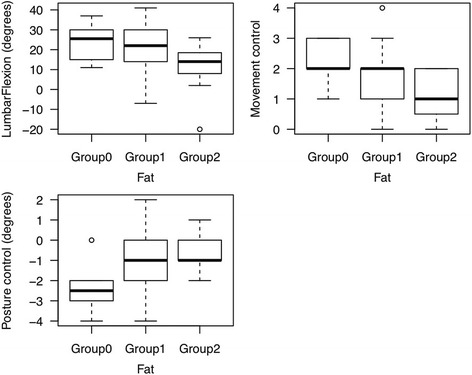
Boxplots showing the association between the severity of fat infiltration (Grade 0, 1 and 2) and lumbar flexion, movement control and posture control. Only decreased lumbar flexion correlated significantly with increased severity of fat infiltration in multifidus muscles. No significant association was found between the severity of fat infiltration and impaired movement control or impaired posture control

No significant correlation could be demonstrated between the severity of fat infiltration and movement control and there was no significant correlation found between fat infiltration and posture control (Fig. 4). But patients’ posture control was affected by the duration of LBP. Patients with acute LBP demonstrated a significant (*p* = 0.003) greater lumbar evasive movement in extension than patients with chronic LBP. The ODI scores and the TPD values did not correlate with the severity of fat infiltration in lumbar multifidus muscles.

## Discussion

The main result of our study was that increased severity of fat infiltration in the lumbar multifidus muscles correlated with decreased flexion range of motion of the lumbar spine. Although none of the effect moderators affected our main outcome, our study agreed with the existing evidence about the correlation between fat infiltration in LMM and age [35, 36], gender [17, 36], duration of pain [8] and BMI [15, 19].

In contrast to our hypothesis, we found no significant correlation between the severity of fat infiltration in LMM and impaired movement control or impaired posture control. Furthermore, correlation between fat infiltration of LMM and impaired body awareness or impaired self-assessed functional disability could not be demonstrated.

### Decreased lumbar flexion

The relationship between spinal range of motion and disability in patients with low back pain has already been examined by various authors. Some found no significant correlation between lumbar flexion and reported disability [37, 38], while others reported decreased hip flexion and reduced spinal range of motion in all directions. The latter was found only in patients with LBP and limited straight leg raise [39]. The results of our study demonstrated that fat grade correlated with decrease of lumbar flexion only, whereas the amount of hip flexion increased, albeit not significantly. Therefore the increase of hip flexion might reflect a compensation for the loss of lumbar flexion. To explain the observed reduction in lumbar flexion, possible factors influencing spinal stability have to be highlighted. Shin et al. [40] demonstrated in their work that healthy subjects with the greatest lumbar flexibility had the highest activity levels of the LMM. Panjabi [41] described a model for spinal stability consisting of three subsystems (spinal column, spinal muscles and neural control unit) which together create optimal spinal flexibility and dynamic stability. As these systems are interdependent, one system could compensate for the deficits of another. If LBP occurs, inhibition of neural control is the consequence. Inhibition impedes alpha motor neuron activity in the anterior horn of the spinal cord and inhibits activity of LMM [12]. Thus, lumbar muscles cannot administrate their function anymore, which most likely debilitates postural control. Ongoing pain inhibition leads to alterations in neuro-muscular control, even after remission of LBP [10]. This mechanism becomes chronic and may result in atrophy of LMM. As important stabilizers, LMM act to maintain optimal joint forces, not only in the neutral zone of the spine, but also during prolonged flexion [42]. Therefore, atrophy in the muscular subsystem can lead to instability that must be compensated.

Superficial paraspinal and trunk muscles may have the ability to compensate for the deficit of LMM. Cholewicki et al. [43] investigated the stabilizing function of trunk flexor and extensor muscles in a neutral spine position. They demonstrated that active spinal stability was provided by flexor and extensor coactivation, but participants used different muscle recruitment strategies to achieve lumbar stability. The coactivation of local stabilizers such as LMM and M. tranversus abdominis increase intervertebral stiffness and allow superficial muscles to perform spinal movement. It has been hypothesized that recruitment strategies change in patients with LBP and global muscles try to compensate by global coactivation [44]. Although global coactivation increases stability, it also restricts spinal motion and function. Chan et al. [45] identified changed patterns of elasticity and cross-sectional area in LMM in relation to posture. In upright 25 and 45° forward stooping positions, the multifidus stiffness was higher in LBP patients than that in asymptomatic controls. There is also evidence that altered muscle activation strategies increase trunk stiffness in resting upright postures in recurrent LBP patients [6].

However defined, it cannot be determined whether reduced flexion range of motion of the lumbar spine is a cause or a result of fat infiltration of LMM. Referring to the results of our study, we hypothesize that once muscle activation strategies have changed, fatty infiltration of LMM proceeds. As a consequence, soft and ligamentous tissues that determine lumbar flexion are stiffening to compensate for the loss of dynamic stability. According to O’Sullivan [46], patients with LBP can be subgrouped into different movement dysfunction patterns. Patients with flexion patterns are probably the ones who have a dysfunction of their dorsal stabilizers. Potentially, fatty infiltrations of LMM is only present in the subgroup of patients with flexion patterns. The sample size of our study was too small to subgroup, but for future studies it would be worth trying to determine if there is a trend when subgrouping the patients. Lumbar pathology seen on MRI can play an important role in recurrence of LBP [47]. Whether decreased lumbar flexion and fat infiltration of LMM are risk factors for a recurrence of LBP remain unclear and has to be investigated.

### Association between fat infiltration of LMM and movement control, postural control, body awareness and self-assessed functional disability

In contrast to our hypothesis we found no significant correlation between the grade of fat infiltration and impaired movement control or impaired postural control.

Likewise, there was no significant correlation found between LBP and impaired movement control and body awareness. This contradicts the findings of other authors [3, 48] but can be explained by the rather small sample size and the fact that patients in this study were not subgrouped according to their LBP specificity. Interestingly, fat infiltration in LMM did not affect postural control, but patients with acute LBP demonstrated significantly higher impairment of posture control than patients with chronic LBP. These findings can be explained by using current evidence of the role of LMM in spinal stability and control. Multifidus muscles are predominantly occupied by muscle fiber type one [49] characterized by an extremly high cross-sectional area with very short muscle fibers that produce large forces over a narrow range of length [42]. The part that contributes the most to spinal stabilization is the deepest and also has a greater percentage of type 1 muscle fibers than the superficial part [50]. Ongoing loss of neural influence and mechanical loading leads to the atrophy of muscle fiber type I [51] whereas age-dependent atrophy of skeletal muscles affects predominately type two fibers [35]. The appearance of acute LBP changes corticomotor excitability [52] and first inhibits the deepest part of LMM which accordingly debilitates postural control more than movement control. This might explain our finding that patients with acute LBP demonstrated significantly higher impairment of posture control than patients with chronic LBP. Nevertheless, a correlation between duration of acute or chronic LBP and decreased lumbar flexion could not be demonstrated. In summary, our results revealed that fat infiltration of LMM has little impact on the measured functions of the lower back muscles.

### Limitations

The results presented in this study should be considered cautiously because of the small sample size and the possible methodological bias of a single-center study with only one tester and one reviewer. Unfortunately, we had to accommodate the fact that patients for this study were referred from different healthcare centers and therefore patients’ MRI were generated in different radiology centers. Differing MRI parameters and visual analysis are limiting factors for grading the amount of fat infiltration in LMM and the authors are aware of the possible inaccuracy of the MRI methodology. Standardized MRI protocols and quantitative MRI-based methods should be used in future studies. Likewise, the selection of patients should be refined in order to harmonize the physical characteristics of the cohort. For financial reasons, a control group with no fat infiltration of LMM and without LBP could not be included. The duration of LBP was measured, but the reoccurrence-rate of LBP was not evaluated. Although lumbar flexion was measured with a reliable instrument, other limiting factors for lumbar flexion (fear avoidance, straight leg raise etc.) were not evaluated in this study and should be considered in continuing investigations.

## Conclusion

Fat infiltration in LMM can be found both in acute or chronic LBP patients and in healthy subjects and therefore is not a pain-specific peculiarity. The presented study is the first that investigated the relationship between the severity of fat infiltration in LMM and the severity of lumbar dysfunction. The main result of this study was, that increased severity of fat infiltration in LMM correlated significantly with decreased range of motion of lumbar flexion. Neither the duration of pain, nor age, gender or BMI had an effect on this correlation. Moreover, the severity of fat infiltration in LMM did not correlate with altered movement control, posture control, body awareness and self-assessed functional disability. In summary, this cross-sectional study revealed that fat infiltration of LMM impaires more the flexibility of the lower spine than it affects active functions of the lower back muscles. Whether reduced flexion range of motion of the lumbar spine is a cause or a result of fat infiltration of LMM could not be identified with this study. And it is still not clear if fat infiltration of LMM is a prognostic factor and if patients with LBP and fat infiltration of LMM have to be subgrouped and need special treatment strategies. And last but not least, it has to be investigated whether asymptomatic subjects with decreased lumbar flexion also demonstrate increased fat infiltration of LMM. Further research is necessary to provide evidence whether specific strategies are effective for the treatment of LBP and for the prevention of progressive fat infiltration of LMM.

## Abbreviations

BMI: Body-Mass-Index
CLBP: Chronic Low Back Pain
LBP: Low Back Pain
LMM: Lumbar Multifidus Muscles
MCT: Movement Control Tests
ODI: Oswestry Disability Index
TPD: Two Point Discrimination
